# 2-Amino-5-chloro­pyridinium hydrogen succinate

**DOI:** 10.1107/S1600536810002990

**Published:** 2010-01-30

**Authors:** Madhukar Hemamalini, Hoong-Kun Fun

**Affiliations:** a X-ray Crystallography Unit, School of Physics, Universiti Sains Malaysia, 11800 USM, Penang, Malaysia

## Abstract

In the title salt, C_5_H_6_ClN_2_^+^·C_4_H_5_O_4_^−^, the pyridine N atom is protonated. The pyridinium and amino groups associate *via* a pair of N—H⋯O hydrogen bonds to the carboxyl­ate O atoms of the singly deprotonated succinate anion. The hydrogen succinate anions self-assemble *via* O—H⋯O hydrogen bonds into chains along the *b* axis. The crystal structure is further stabilized by additional N—H⋯O hydrogen bonds involving the second amino H atoms, as well as C—H⋯O contacts, forming a three-dimensional network.

## Related literature

For background to the chemistry of substituted pyridines, see: Pozharski *et al.* (1997 ▶); Katritzky *et al.* (1996 ▶). For related structures, see: Pourayoubi *et al.* (2007 ▶); Akriche & Rzaigui (2005 ▶); Zaouali Zgolli *et al.* (2009 ▶). For the structure of succinic acid, see: Gopalan *et al.* (2000 ▶); Leviel *et al.* (1981 ▶). For applications of succinic acid, see: Sauer *et al.* (2008 ▶); Song & Lee (2006 ▶); Zeikus *et al.* (1999 ▶)·For details of hydrogen bonding, see: Jeffrey & Saenger (1991 ▶); Jeffrey (1997 ▶); Scheiner (1997 ▶). For hydrogen-bond motifs, see: Bernstein *et al.* (1995 ▶). For the stability of the temperature controller used in the data collection, see: Cosier & Glazer (1986 ▶).
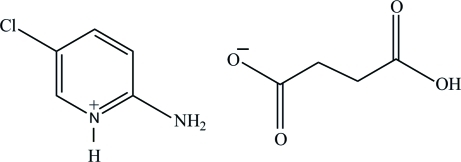


## Experimental

### 

#### Crystal data

C_5_H_6_ClN_2_^+^·C_4_H_5_O_4_^−^
*M* *_r_* = 246.65
Orthorhombic, 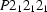

*a* = 5.2263 (1) Å
*b* = 13.5997 (3) Å
*c* = 14.9019 (3) Å
*V* = 1059.17 (4) Å^3^
*Z* = 4
Mo *K*α radiation
μ = 0.36 mm^−1^
*T* = 100 K
0.41 × 0.15 × 0.10 mm

#### Data collection

Bruker SMART APEXII CCD area-detector diffractometer
Absorption correction: multi-scan (*SADABS*; Bruker, 2009 ▶) *T*_min_ = 0.866, *T*_max_ = 0.965
11594 measured reflections
3934 independent reflections
3581 reflections with *I* > 2σ(*I*)
*R*_int_ = 0.033

#### Refinement

*R*[*F* ^2^ > 2σ(*F* ^2^)] = 0.038
*wR*(*F* ^2^) = 0.082
*S* = 1.02
3934 reflections
189 parameters
H atoms treated by a mixture of independent and constrained refinement
Δρ_max_ = 0.33 e Å^−3^
Δρ_min_ = −0.27 e Å^−3^
Absolute structure: Flack (1983 ▶), 1604 Friedel pairs
Flack parameter: 0.05 (5)

### 

Data collection: *APEX2* (Bruker, 2009 ▶); cell refinement: *SAINT* (Bruker, 2009 ▶); data reduction: *SAINT*; program(s) used to solve structure: *SHELXS97* (Sheldrick, 2008 ▶); program(s) used to refine structure: *SHELXL97* (Sheldrick, 2008 ▶); molecular graphics: *SHELXTL* (Sheldrick, 2008 ▶); software used to prepare material for publication: *SHELXTL* and *PLATON* (Spek, 2009 ▶).

## Supplementary Material

Crystal structure: contains datablocks global, I. DOI: 10.1107/S1600536810002990/tk2616sup1.cif

Structure factors: contains datablocks I. DOI: 10.1107/S1600536810002990/tk2616Isup2.hkl

Additional supplementary materials:  crystallographic information; 3D view; checkCIF report

## Figures and Tables

**Table 1:** Hydrogen-bond geometry (Å, °)

*D*—H⋯*A*	*D*—H	H⋯*A*	*D*⋯*A*	*D*—H⋯*A*
O3—H1*O*3⋯O2^i^	0.821 (19)	1.819 (19)	2.5891 (15)	156 (2)
N1—H1*N*1⋯O2^ii^	0.86 (2)	1.85 (2)	2.7023 (15)	172.4 (19)
N2—H1*N*2⋯O1^ii^	0.84 (2)	1.95 (2)	2.7814 (15)	177 (2)
N2—H2*N*2⋯O1	0.826 (19)	2.004 (19)	2.8002 (16)	162 (2)
C5—H5⋯O4^iii^	0.964 (17)	2.391 (17)	3.2216 (18)	144.0 (13)

Symmetry codes: (i) 
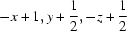
; (ii) 
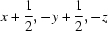
; (iii) 
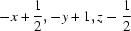
.

**Fig. 1.**
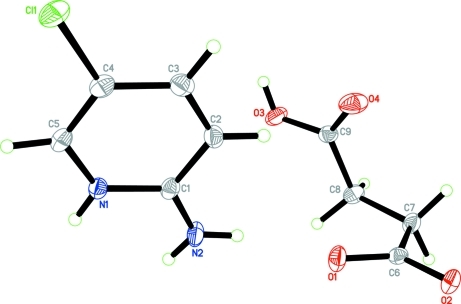
The asymmetric unit of (I) showing atom labelling scheme. Displacement ellipsoids are drawn at the 50% probability level.

**Fig. 2.**
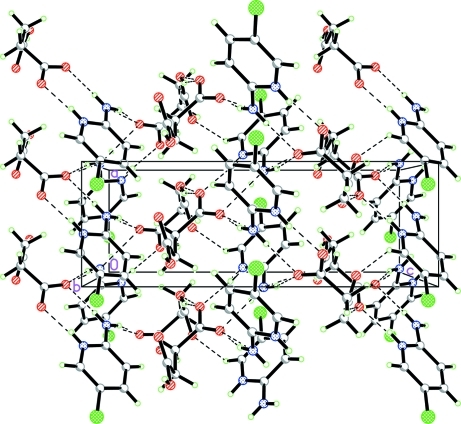
The crystal packing of (I), showing intermolecular interactions as dashed lines.

## supplementary crystallographic information

### Comment

Pyridine and its derivatives play an important role in heterocyclic chemistry (Pozharski *et al.*, 1997; Katritzky *et al.*, 1996). They are often involved in hydrogen-bonding interactions (Jeffrey & Saenger, 1991; Jeffrey, 1997; Scheiner, 1997). The dicarboxylic acid, succinic acid, is a precursor for many chemicals of industrial importance (Zeikus *et al.*, 1999; Song & Lee, 2006). Succinic acid derivatives are mostly used in chemicals, food and pharmaceuticals (Sauer *et al.*, 2008). The crystal structure of succinic acid has been reported (Gopalan *et al.*, 2000; Leviel *et al.*, 1981). The crystal structures of 2-amino-5-chloropyridine (Pourayoubi *et al.*, 2007), 2-amino-5-chloropyridinium nitrate (Zaouali Zgolli *et al.*, 2009) and bis (2-amino-5-chloropyridinium) dihydrogen diphosphate (Akriche & Rzaigui, 2005) have been reported in literature. In this paper, we present the X-ray single-crystal structure of 2-amino-5-chloropyridinium hydrogen succinate, (I).

The asymmetric unit of (I), Fig. 1, contains a 2-amino-5-chloropyridinium cation and a hydrogen succinate anion, indicating that proton transfer has occurred during the co-crystallisation experiment. In the 2-amino-5-chloropyridinium cation, a wider than normal angle (123.22 (12)°) is subtended at the protonated N1 atom.

In the crystal packing (Fig. 2), the protonated N1 atom and the 2-amino group is hydrogen-bonded to the carboxylate oxygen atoms (O1 and O2) via a pair of N–H···O hydrogen bonds forming a *R*_2_^2^(8) ring motif (Bernstein *et al.* 1995). The hydrogen succinate anions self-assemble via O—H···O hydrogen bonds. The second amino-H atom forms a hydrogen bond with the carboxylate-O1 atom. Furthermore, the crystal structure is stabilized by C—H···O contacts, Table 1, forming a 3D-network.

### Experimental

A hot methanolic solution (10 ml) of 2-amino-5-chloropyridine (32 mg, Aldrich) and a hot aqueous solution (10 ml) of succinic acid (29 mg, Merck) were mixed and warmed over a water bath for 10 minutes. The resulting solution was allowed to cool slowly at room temperature. Single crystals of (I) appeared from the mother liquor after a few days.

### Refinement

All the H atoms were located in a difference Fourier map and allowed to refine freely [N–H = 0.83 (2) - 0.86 (2) Å, C–H = 0.944 (18) - 1.047 (19) Å, O–H = 0.822 (19) Å ].

### Crystal data

**Table:**

C_5_H_6_ClN_2_^+^·C_4_H_5_O_4_^−^	*F*(000) = 512
*M**_r_* = 246.65	*D*_x_ = 1.547 Mg m^−^^3^
Orthorhombic, *P*2_1_2_1_2_1_	Mo *K*α radiation, λ = 0.71073 Å
Hall symbol: P 2ac 2ab	Cell parameters from 3633 reflections
*a* = 5.2263 (1) Å	θ = 2.7–33.2°
*b* = 13.5997 (3) Å	µ = 0.36 mm^−^^1^
*c* = 14.9019 (3) Å	*T* = 100 K
*V* = 1059.17 (4) Å^3^	Blcok, yellow
*Z* = 4	0.41 × 0.15 × 0.10 mm

### Data collection

**Table:**

Bruker SMART APEXII CCD area-detector diffractometer	3934 independent reflections
Radiation source: fine-focus sealed tube	3581 reflections with *I* > 2σ(*I*)
graphite	*R*_int_ = 0.033
φ and ω scans	θ_max_ = 33.2°, θ_min_ = 2.0°
Absorption correction: multi-scan (*SADABS*; Bruker, 2009)	*h* = −8→6
*T*_min_ = 0.866, *T*_max_ = 0.965	*k* = −17→20
11594 measured reflections	*l* = −21→22

### Refinement

**Table:**

Refinement on *F*^2^	Secondary atom site location: difference Fourier map
Least-squares matrix: full	Hydrogen site location: inferred from neighbouring sites
*R*[*F*^2^ > 2σ(*F*^2^)] = 0.038	H atoms treated by a mixture of independent and constrained refinement
*wR*(*F*^2^) = 0.082	*w* = 1/[σ^2^(*F*_o_^2^) + (0.0404*P*)^2^ + 0.0588*P*] where *P* = (*F*_o_^2^ + 2*F*_c_^2^)/3
*S* = 1.02	(Δ/σ)_max_ = 0.001
3934 reflections	Δρ_max_ = 0.33 e Å^−^^3^
189 parameters	Δρ_min_ = −0.27 e Å^−^^3^
0 restraints	Absolute structure: Flack (1983), 1604 Friedel pairs
Primary atom site location: structure-invariant direct methods	Flack parameter: 0.05 (5)

### Special details

**Table:**

Experimental. The crystal was placed in the cold stream of an Oxford Cryosystems Cobra open-flow nitrogen cryostat (Cosier & Glazer, 1986) operating at 100.0 (1) k.
Geometry. All esds (except the esd in the dihedral angle between two l.s. planes) are estimated using the full covariance matrix. The cell esds are taken into account individually in the estimation of esds in distances, angles and torsion angles; correlations between esds in cell parameters are only used when they are defined by crystal symmetry. An approximate (isotropic) treatment of cell esds is used for estimating esds involving l.s. planes.
Refinement. Refinement of F^2^ against ALL reflections. The weighted R-factor wR and goodness of fit S are based on F^2^, conventional R-factors R are based on F, with F set to zero for negative F^2^. The threshold expression of F^2^ > 2sigma(F^2^) is used only for calculating R-factors(gt) etc. and is not relevant to the choice of reflections for refinement. R-factors based on F^2^ are statistically about twice as large as those based on F, and R- factors based on ALL data will be even larger.

### Fractional atomic coordinates and isotropic or equivalent isotropic displacement parameters (Å^2^)

**Table:**

	*x*	*y*	*z*	*U*_iso_*/*U*_eq_	
Cl1	−0.16471 (6)	0.66417 (3)	0.01385 (3)	0.02344 (8)	
N1	0.3976 (2)	0.47974 (9)	−0.04334 (8)	0.0152 (2)	
N2	0.5510 (2)	0.33552 (9)	0.01762 (9)	0.0194 (2)	
C1	0.3838 (2)	0.40861 (10)	0.02044 (9)	0.0152 (2)	
C2	0.1897 (3)	0.41668 (10)	0.08661 (9)	0.0170 (2)	
C3	0.0245 (3)	0.49419 (11)	0.08409 (10)	0.0183 (3)	
C4	0.0469 (2)	0.56603 (10)	0.01630 (10)	0.0180 (2)	
C5	0.2340 (3)	0.55775 (10)	−0.04688 (10)	0.0168 (2)	
O1	0.42571 (19)	0.16607 (8)	0.11491 (6)	0.0208 (2)	
O2	0.24386 (19)	0.03548 (7)	0.17729 (7)	0.0184 (2)	
O3	0.8289 (2)	0.36179 (8)	0.25630 (8)	0.0232 (2)	
O4	0.4478 (2)	0.29887 (8)	0.29328 (8)	0.0277 (3)	
C6	0.4118 (2)	0.10302 (9)	0.17571 (9)	0.0142 (2)	
C7	0.6061 (3)	0.10441 (10)	0.25145 (10)	0.0179 (3)	
C8	0.7907 (2)	0.19017 (10)	0.24860 (10)	0.0181 (3)	
C9	0.6663 (3)	0.28792 (10)	0.26836 (8)	0.0158 (2)	
H2	0.180 (3)	0.3686 (13)	0.1322 (11)	0.020 (4)*	
H3	−0.110 (3)	0.4991 (12)	0.1279 (11)	0.020 (4)*	
H5	0.262 (3)	0.6021 (12)	−0.0964 (12)	0.016 (4)*	
H7A	0.713 (4)	0.0395 (14)	0.2466 (13)	0.029 (5)*	
H7B	0.504 (4)	0.1041 (13)	0.3078 (13)	0.033 (5)*	
H8A	0.925 (4)	0.1818 (13)	0.2917 (12)	0.026 (5)*	
H8B	0.883 (4)	0.1978 (13)	0.1893 (12)	0.023 (5)*	
H1O3	0.764 (4)	0.4144 (14)	0.2709 (13)	0.026 (5)*	
H1N1	0.517 (4)	0.4729 (13)	−0.0823 (13)	0.026 (5)*	
H1N2	0.667 (4)	0.3364 (14)	−0.0209 (13)	0.029 (5)*	
H2N2	0.538 (4)	0.2904 (14)	0.0544 (13)	0.027 (5)*	

### Atomic displacement parameters (Å^2^)

**Table:**

	*U*^11^	*U*^22^	*U*^33^	*U*^12^	*U*^13^	*U*^23^
Cl1	0.01978 (14)	0.01782 (15)	0.03271 (18)	0.00423 (12)	0.00071 (13)	−0.00177 (14)
N1	0.0162 (5)	0.0134 (5)	0.0162 (5)	−0.0010 (4)	0.0012 (4)	0.0015 (4)
N2	0.0200 (5)	0.0154 (5)	0.0227 (6)	0.0009 (5)	0.0046 (5)	0.0071 (5)
C1	0.0156 (5)	0.0133 (5)	0.0168 (6)	−0.0035 (4)	−0.0024 (4)	0.0009 (5)
C2	0.0188 (6)	0.0168 (6)	0.0154 (6)	−0.0038 (5)	0.0007 (5)	0.0013 (5)
C3	0.0177 (6)	0.0193 (6)	0.0180 (6)	−0.0033 (5)	0.0013 (5)	−0.0030 (5)
C4	0.0176 (5)	0.0154 (6)	0.0209 (6)	0.0001 (5)	−0.0026 (5)	−0.0021 (5)
C5	0.0175 (5)	0.0124 (6)	0.0205 (7)	−0.0014 (5)	−0.0030 (5)	0.0008 (5)
O1	0.0257 (5)	0.0174 (5)	0.0194 (5)	−0.0048 (4)	−0.0037 (4)	0.0068 (4)
O2	0.0197 (4)	0.0132 (4)	0.0222 (5)	−0.0027 (4)	−0.0035 (4)	0.0039 (4)
O3	0.0242 (5)	0.0129 (5)	0.0326 (6)	−0.0011 (4)	0.0082 (5)	−0.0048 (4)
O4	0.0192 (5)	0.0251 (6)	0.0387 (6)	−0.0001 (4)	0.0062 (4)	−0.0112 (5)
C6	0.0153 (5)	0.0118 (5)	0.0154 (6)	0.0024 (4)	0.0000 (4)	−0.0013 (5)
C7	0.0226 (6)	0.0140 (6)	0.0169 (6)	−0.0011 (5)	−0.0027 (5)	0.0027 (5)
C8	0.0169 (6)	0.0156 (6)	0.0218 (7)	0.0014 (5)	−0.0023 (5)	−0.0016 (5)
C9	0.0184 (5)	0.0153 (6)	0.0138 (6)	0.0011 (5)	−0.0014 (5)	−0.0017 (5)

### Geometric parameters (Å, °)

**Table:**

Cl1—C4	1.7336 (13)	C5—H5	0.965 (17)
N1—C1	1.3580 (17)	O1—C6	1.2496 (16)
N1—C5	1.3636 (18)	O2—C6	1.2708 (16)
N1—H1N1	0.86 (2)	O3—C9	1.3281 (17)
N2—C1	1.3242 (17)	O3—H1O3	0.822 (19)
N2—H1N2	0.83 (2)	O4—C9	1.2100 (17)
N2—H2N2	0.83 (2)	C6—C7	1.5183 (19)
C1—C2	1.4189 (18)	C7—C8	1.5141 (19)
C2—C3	1.363 (2)	C7—H7A	1.047 (19)
C2—H2	0.944 (18)	C7—H7B	0.99 (2)
C3—C4	1.410 (2)	C8—C9	1.5090 (19)
C3—H3	0.961 (17)	C8—H8A	0.959 (19)
C4—C5	1.362 (2)	C8—H8B	1.010 (18)
			
C1—N1—C5	123.22 (12)	N1—C5—H5	115.0 (10)
C1—N1—H1N1	115.7 (12)	C9—O3—H1O3	110.9 (14)
C5—N1—H1N1	121.0 (12)	O1—C6—O2	123.33 (12)
C1—N2—H1N2	119.3 (14)	O1—C6—C7	119.44 (11)
C1—N2—H2N2	118.7 (13)	O2—C6—C7	117.21 (11)
H1N2—N2—H2N2	122.0 (19)	C8—C7—C6	114.51 (11)
N2—C1—N1	118.52 (12)	C8—C7—H7A	107.9 (10)
N2—C1—C2	123.49 (12)	C6—C7—H7A	107.2 (10)
N1—C1—C2	117.99 (12)	C8—C7—H7B	111.6 (11)
C3—C2—C1	119.57 (13)	C6—C7—H7B	105.6 (12)
C3—C2—H2	121.4 (11)	H7A—C7—H7B	109.9 (15)
C1—C2—H2	119.0 (11)	C9—C8—C7	113.48 (11)
C2—C3—C4	120.21 (13)	C9—C8—H8A	106.9 (11)
C2—C3—H3	119.8 (10)	C7—C8—H8A	110.9 (11)
C4—C3—H3	119.9 (10)	C9—C8—H8B	106.5 (10)
C5—C4—C3	119.81 (12)	C7—C8—H8B	114.0 (10)
C5—C4—Cl1	120.47 (11)	H8A—C8—H8B	104.4 (14)
C3—C4—Cl1	119.71 (11)	O4—C9—O3	123.52 (13)
C4—C5—N1	119.20 (13)	O4—C9—C8	125.11 (13)
C4—C5—H5	125.8 (10)	O3—C9—C8	111.36 (12)
			
C5—N1—C1—N2	179.59 (12)	Cl1—C4—C5—N1	−179.96 (10)
C5—N1—C1—C2	−0.30 (18)	C1—N1—C5—C4	0.21 (19)
N2—C1—C2—C3	−179.60 (13)	O1—C6—C7—C8	4.56 (19)
N1—C1—C2—C3	0.29 (18)	O2—C6—C7—C8	−176.50 (12)
C1—C2—C3—C4	−0.2 (2)	C6—C7—C8—C9	69.12 (16)
C2—C3—C4—C5	0.1 (2)	C7—C8—C9—O4	7.3 (2)
C2—C3—C4—Cl1	179.95 (11)	C7—C8—C9—O3	−173.70 (12)
C3—C4—C5—N1	−0.10 (19)		

### Hydrogen-bond geometry (Å, °)

**Table:**

*D*—H···*A*	*D*—H	H···*A*	*D*···*A*	*D*—H···*A*
O3—H1O3···O2^i^	0.821 (19)	1.819 (19)	2.5891 (15)	156 (2)
N1—H1N1···O2^ii^	0.86 (2)	1.85 (2)	2.7023 (15)	172.4 (19)
N2—H1N2···O1^ii^	0.84 (2)	1.95 (2)	2.7814 (15)	177 (2)
N2—H2N2···O1	0.826 (19)	2.004 (19)	2.8002 (16)	162 (2)
C5—H5···O4^iii^	0.964 (17)	2.391 (17)	3.2216 (18)	144.0 (13)

Symmetry codes: (i) −*x*+1, *y*+1/2, −*z*+1/2; (ii) *x*+1/2, −*y*+1/2, −*z*; (iii) −*x*+1/2, −*y*+1, *z*−1/2.
